# Evaluation of DC/DC switching power regulation with small-scale integrated inductors for PET/MR

**DOI:** 10.1186/2197-7364-2-S1-A14

**Published:** 2015-05-18

**Authors:** Laura Biagi, Maria Giuseppina Bisogni, Niccolo Camarlinghi, Mauro Costagli, Giancarlo Sportelli, Michela Tosetti, Alberto Del Guerra, Nicola Belcari

**Affiliations:** Department of Physics, University of Pisa and INFN, Pisa, Italy; IRCCS Fondazione Stella Maris and Fondazione Imago 7, Calambrone, Pisa Italy

We present a feasibility study that has been carried out to determine the best power regulation strategy for the PET front-end electronics of the trimodal PET/MRI/EEG TRIMAGE scanner. Conventional power regulation strategies cannot be applied to PET/MRI because standard switching regulators stop working in presence of a high magnetic field. At the state of the art, linear regulators are used instead. However, linear regulators are inefficient and might not allow to fulfill power and thermal constraints if the electronics becomes more power demanding, such as in the case of FPGA based front-ends. Very recently, a new generation of switching power supplies has been introduced for EMI critical applications where the discrete inductor energy buffer is not allowed. These supplies have very small footprint, need few biasing peripherals and they use on-chip integrated inductors for energy storage. These switching power regulators coupled with an adequate EMI shield could be an achievable power solution for our PET front-end electronics. Test procedures for Enpirion. EN2390QI and the Enpirion. EN6347QI switching power regulators are presented. Measurements have been performed at GE 1.5T MRI scanner with the support of IRCCS Fondazione Stella Maris. All the board have been tested in two different configurations: with and without an additional EMI shield. Performance of these two switching power regulators have been compared with a linear power regulator (Enpirion. EY1501DI). Output voltage, output current and temperature have been measured. The stability of these three main characteristic will be presented in different operation conditions and will be discussed (output voltage vs. temperature, output voltage vs. output current and output current vs. temperature).

